# Inclusive Co-Design of Augmented Reality Digital Helpers for Autistic Adolescents and Young Adults: A Study on Just-In-Time Augmented Reality Assistance

**DOI:** 10.1177/29941520251380700

**Published:** 2025-10-06

**Authors:** Julie E. LeMoine, Jean A. Frazier, Benjamin Dadagian-Goldman, Lizbeth Goodman, Kevin Marshall

**Affiliations:** ^1^ UMass Chan Medical School, Worcester, Massachusetts, USA.; ^2^ SMARTlab-IDRC, College of Engineering and Architecture, University College Dublin, Dublin, Ireland.; ^3^ Microsoft EMEA, County Dublin, Dublin, Ireland.

**Keywords:** autism, Transition Age Youth, adolescents and young adults, augmented reality, life challenges, assistive technology, inclusive design

## Abstract

This study explored inclusive, augmented reality (AR) digital helpers as assistive technology that are codesigned by autistic adolescents and young adults. The study used a participatory design process and partnered with autistic adolescents and young adults to codesign AR digital helper prototypes. The findings indicated a positive impact from the codesign role for autistic participants and high enthusiasm for applied AR digital helpers from all participant groups. The study established new insights into AR digital helper characteristics and their perceived utility as helpers for autistic adolescents and young adults. This article presents a summary of the study’s methods, discusses its findings and limitations, and outlines potential future research goals.

## Introduction

In the United States, 1 in 36 children is autistic,^1^ and over 620,000 autistic children are born each year.^1,2^ As these children grow up, they face limited access to critical services that support independent living. This is due to funding constraints and a growing shortage of trained mental health professionals.

Autistic adolescents and young adults, also known as Transition Age Youth (TAY), face more and different challenges than their neurotypical peers, especially as they age out of pediatric support systems and enter adulthood. According to the DSM-5,^3^ autistic youth often struggle with time management, turning in assignments, traveling, personal safety, social skills, dealing with change, and self-advocacy. Life goals such as graduating from high school, attending college, maintaining employment, managing money, staying healthy, and building social relationships add further challenges.^1–3^

Extended reality (XR) includes immersive technologies like virtual reality (VR) and augmented reality (AR). VR provides full or near-full immersion in a simulated environment, usually through a head-mounted display.^4,5^ In contrast, AR overlays digital content—such as images, animations, or videos—onto the real world. AR provides this added layer to the real world for users who view the world through devices with cameras that are able to run AR applications (e.g., these AR glasses, smartphones, and some tablets).^5^

Existing autism research using immersive technologies has mainly used VR. The VR use cases focused primarily on understanding VR’s potential to promote learning, build empathy, or study autistic individuals.^6–12^ There have been approximately one-quarter as many studies on the use of AR compared with VR in autism spectrum disorder (ASD) research.^13,14^ These studies continue to focus primarily on learning and skill development rather than addressing everyday challenges and personal goals.

This study introduced AR-based digital helpers designed for autistic TAYs.^15^ These helpers have the potential to provide Just-In-Time (JIT) assistance with everyday life challenges and life goals. The study aimed to (1) identify the most and least important characteristics of AR digital helpers for autistic TAY, and (2) determine which daily challenges and goals these helpers should address. It also gathered feedback on the value of codesign and on participants’ concerns and hopes for this type of support. This research was guided by principles from inclusive design theory (IDT),^16–18^ critical disability theory,^19–21^ and self-efficacy theory related to IDT.^22,23^

This early-stage research codesigned AR helper characters with its participating autistic TAY. Based on these codesigns, it created novel JIT digital helpers. The codesign process and resulting AR digital helpers, filmed in action offering assistance, were used to gather data about critical characteristics of the helpers. The study also gathered data on the everyday challenges with which such helpers should offer assistance. These latter data were collected from participating autistic TAY, members of their care ecosystem, and independent experts in the field of autism support services and research. Finally, data were collected from all participants on the importance of codesigning, how useful these helpers might be for autistic TAYs, and if these types of helpers would be “good” (healthy and enjoyable) for autistic TAYs.

## Methods

This cross-sectional study included autistic TAY, members of their care ecosystems, and independent autism care providers. A total of 79 participants took part in the study. These included 20 autistic TAY, with self- or parent-reported IQ over 70—10 adolescents aged 14–17 and 10 young adults aged 18–22. Each TAY participant was required to have 1 parent or guardian involved (*N* = 20), and 15 had an optionally involved care provider (Inv-CP). Additionally, 24 independent care providers (Ind-CP), who were not directly involved with the participating TAY, contributed an external perspective. All participating parents or guardians were parents of the TAY. All participants also needed to be able to read and respond to surveys and watch videos in English. Due to COVID-19 restrictions, in-person visits were redesigned as online sessions. Two study protocols were developed and used. Both the TAY and one parent or guardian had to attend two online study visits. Inv-CPs and Ind-CPs were not required to attend live study visits. The Inv-CPs and Ind-CPs participated by responding to a single questionnaire via an online questionnaire.

Recruitment was conducted through publicly available channels. These included autism research bulletin boards, online discussion forums, email flyers sent to interest groups, and physical flyers posted in pediatric service areas near the UMass Chan Eunice Kennedy Shriver Center. Clinicians familiar with the research also shared the study by word of mouth. Because the study was conducted online, participants could be located anywhere in the United States. Most were based in the eastern states, including Massachusetts, New York, New Jersey, and Pennsylvania.

The study and its Data Collection Protocols (DCPs) were reviewed and approved by the ethics committees at University College Dublin (Study # LS-19-48-LeMoine-Goodman) and UMass Chan Medical School (IRB # H00018214_1). The study used REDCap to design and distribute online questionnaires and to collect participant data securely. The questionnaires used a mixed-methods design with both quantitative and qualitative items. They included Likert-scale and multiple-response questions, as well as open-ended prompts. Adaptive routing was used to tailor the flow of questions based on participant responses for the codesign sessions. The latter reduced the number of questions and improved relevance for participating TAY. All other data were collected using fixed-order, mixed-method questionnaires.

All participants had the opportunity to view a video of an AR Digital Helper providing assistance. TAY participants viewed their own codesigned helpers in action. All other participants viewed the same standardized digital helper to ensure consistency.

Quantitative data were analyzed descriptively. Frequencies and percentages were calculated to summarize responses. No inferential statistics or group comparisons were conducted. This approach aligns with IDT, which emphasizes participant voice, diversity of experience, and co-creation of knowledge over generalization or comparison.^16–18^

**FIG. 1.**
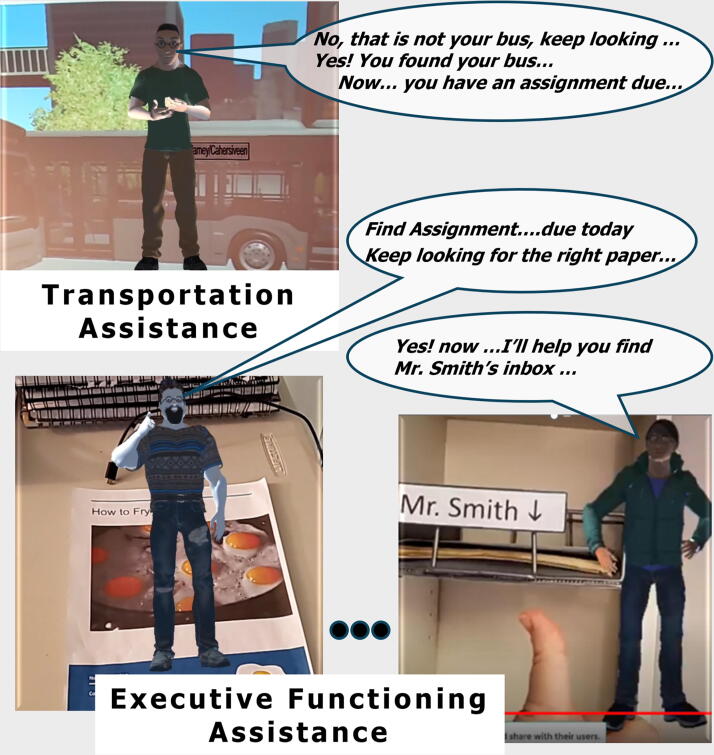
Live screen snaps of study’s AR digital helpers in action. AR, augmented reality.

Twenty AR digital helpers were codesigned with autistic TAY. One additional helper was created for caregiver participants. Each helper supported three assistive activities. The helper helped the user find the right bus, supporting travel independence and wayfinding. Then the helper identified a currently due assignment and guided the user to the right inbox to submit the assignment. The latter two tasks fall into both executive functioning and wayfinding support. These activities were selected from research-based life challenges commonly faced by autistic TAY. The research selected three tasks in an effort to demonstrate the broad potential of AR digital helpers. Each helper spoke to and guided users through these tasks. Figure 1 shows screenshots from 3 of the 20 codesigned AR digital helper applications, each assisting with one of the supported activities.

Qualitative data were analyzed using inductive thematic analysis. Responses to each open-ended question were reviewed to identify recurring ideas and patterns. Themes were developed per question, grounded in the participants’ own language and perspectives. This method supported the study’s inclusive and participatory design goals by centering the voices of autistic TAY and their caregivers.^19–26^

Initial demographic data were collected from each TAY and their participating parent or guardian. These data included the TAY’s age, gender, education level, transportation experience, 3D gaming experience, and prior exposure to VR and AR. Participants also reported their life challenges and goals. Two DCPs were defined and used: the Characteristics DCP and the Value Proposition DCP. Activities from both protocols were conducted during the two online study visits attended by each TAY and their parent participant. The Inc-CP and the Ind-CP participated in the Value Proposition DCP via a single online questionnaire.

The Characteristics DCP captured codesign input from each TAY about their desired digital helper. This included preferences for 27 specific features across 11 major categories. During the first study visit, a semistructured interview was used to gather these preferences. The researcher used this input to guide the creation of each TAY’s AR digital helper, establishing the TAY as a codesigner.

Between the first and second visits, the researcher created each TAY’s digital helper and developed a marker-based AR prototype application for Android. The prototype was built using over 15 tools, including Unity, Vuforia, custom C# code, Reallusion Character Creator, iClone, Blender, and Audacity.^15^ Each AR helper application was deployed and filmed in action on the Vuzix Blade AR glasses and the Android smartphones.

At the second study visit, each TAY participant viewed a video of their own, codesigned AR digital helper in action. After viewing their helper in action, they were asked which of the 11 characteristic categories were important in their helper. They were also asked if they would change any feature settings or suggest improvements. This two-visit process led to the development of a new participatory design methodology called Partnership Design©.^15^ This method adds emphasis on having researchers share the knowledge needed for participation without influencing or levying unrealistic learning burdens for participants. A follow-up article on this methodology is planned.

The Value Proposition DCP focused on identifying JIT assistive activities that AR digital helpers should support. Before viewing the AR helper, TAY and their parents completed surveys about their life challenges and goals. After viewing a video of an AR digital helper in action, all participants, including Inv-CPs and Ind-CPs, were asked which assistive activities the helper should support.

**FIG. 2.**
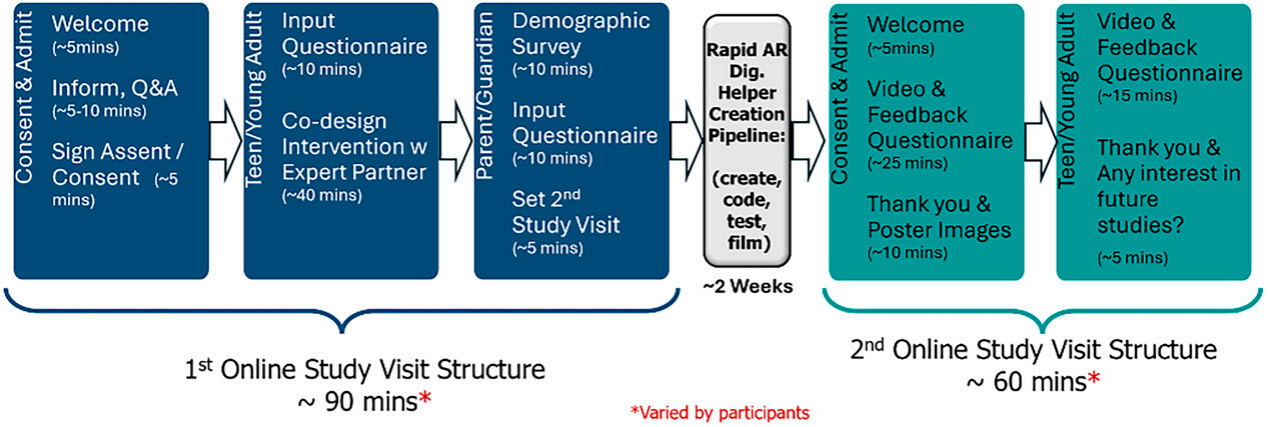
Per TAY participant, study visits activity flow. TAY, Transition Age Youth.

The flow of each online, in-person study visit with each TAY and one parent is summarized in Figure 2.

The overall study ran from 2019 to 2022. In the first 18 months, the technical framework needed to establish each codesigned helper within a 2-week goal was established. Recruitment and study visit activities lasted 6 months. Seventeen TAY groups completed their sessions in the first 3 months. The data collection questions used during the second study visit included many from the first study visit. This allowed the study to see how participants adjusted their responses after seeing their digital helper in action.

## Results

The study collected more than 4,000 data assets through qualitative and quantitative questionnaires. These focused on AR helper characteristics and areas of assistance. Due to the wide spectrum of autism and the study’s inclusive design goals, standard deviation calculations were excluded from the analysis. Instead, the quantitative analysis emphasized the most and least selected responses.

Qualitative data were coded for thematic analysis. Responses from TAY participants, their care ecosystems, and independent care providers showed strong interest in AR digital helpers. All participants supported codesigning their own helper as a way to promote engagement, confidence, and personal agency. Key findings from this study’s two main research questions are presented next, followed by results related to three subquestions.

### Results aim 1: Key characteristics

**FIG. 3.**
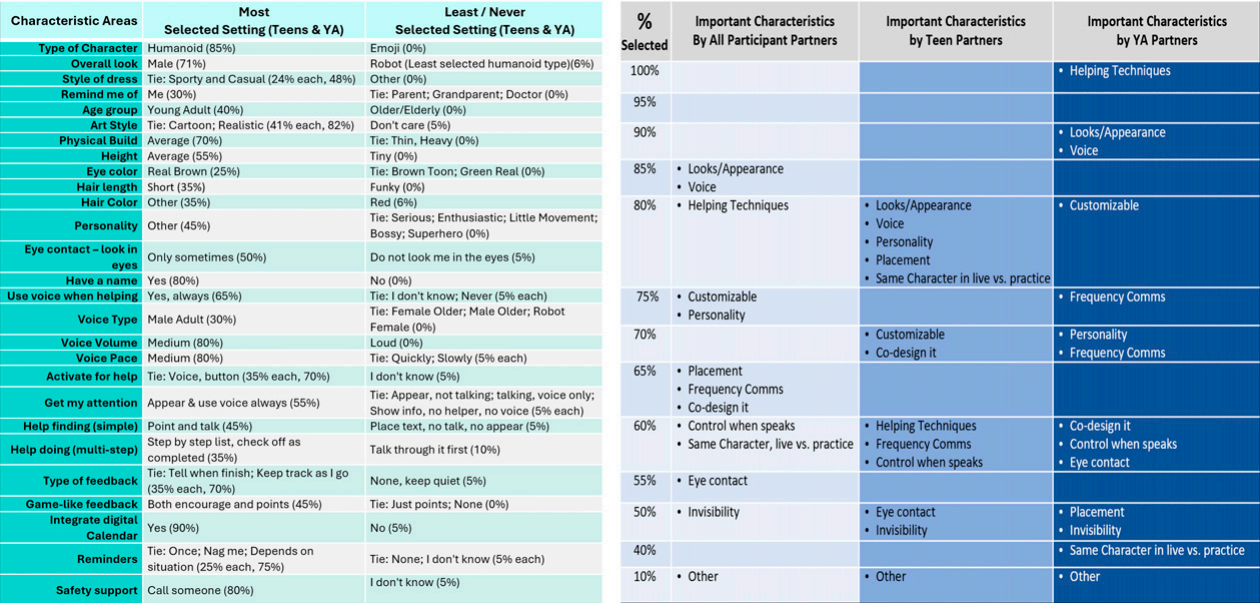
Most vs. least selected settings for characteristics by adolescent vs. young adults.

These data were collected during the first study visit by asking TAY participants to define their preferred AR digital helper features across 27 areas. Only TAY participants contributed to this dataset. These data were used to inclusively design each helper. Figure 3 presents these findings and compares responses between adolescents and young adults in two formats.

After seeing their codesigned helper in action (second study visit), all TAY participants preferred a visible character over a voice-only assistant, such as Alexa©. Although the study emphasized that helpers could be voice-only or take any form, including animals or emojis, 85% chose humanoid characters. In qualitative responses, many shared that human-like helpers made it easier to understand emotional cues and interactions. Furthermore, 70% of those choosing humanoid helpers requested a specific person or character as their helper. The remaining 30% asked for a version of themselves.

Young adult participants (ages 18–22) gave high ratings to how the digital helper offered assistance. In related qualitative responses, many described the helper as a potential “virtual friend.” Ninety percent of TAY asked for calendar integration to support daily tasks. In the gamification category, 100% wanted their helper to offer encouragement. Some also requested points for completing tasks.

All participants contributed to identifying which of the 11 characteristic areas were most important. Interestingly, TAY participants gave low ratings to whether others could see that they were receiving help (invisibility) or whether they could control the helper’s eye contact. These were expected to be important by the study team but ranked lowest overall.

**FIG. 4.**
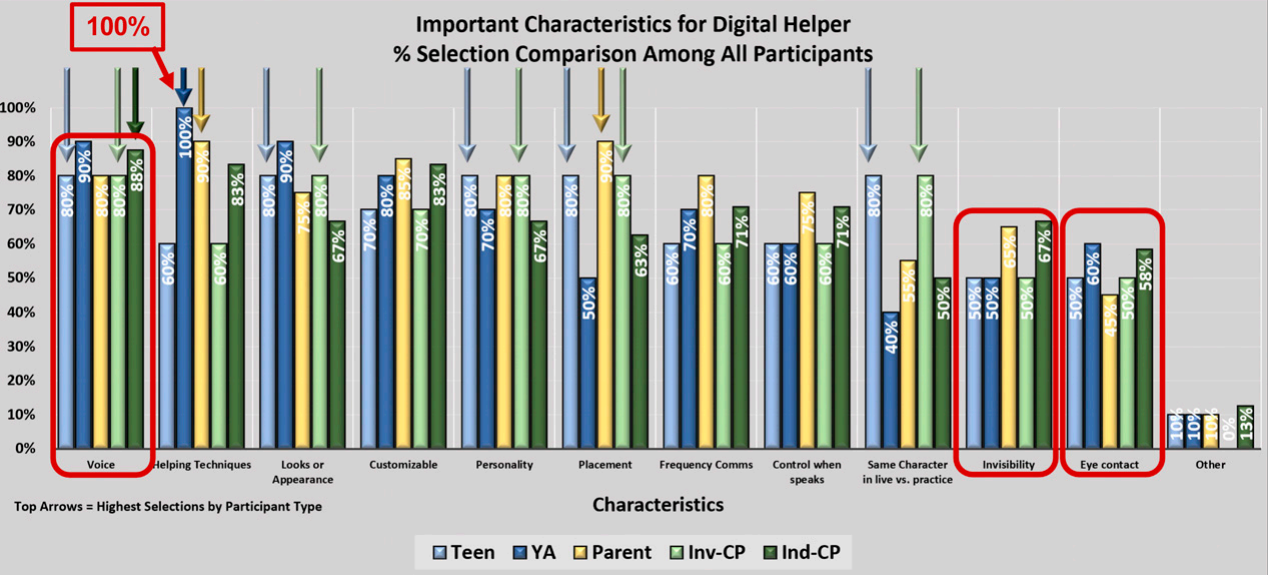
Most and least selected as important characteristics area by each participant type.

Figure 4 summarizes these results and shows the percentage of each participant group that selected each area. Among the 11 core categories, voice, helping techniques, and appearance ranked highest. Customizable settings like calendar integration and visibility control were also valued.

### Results aim 2: Life challenges and life goals

**FIG. 5.**
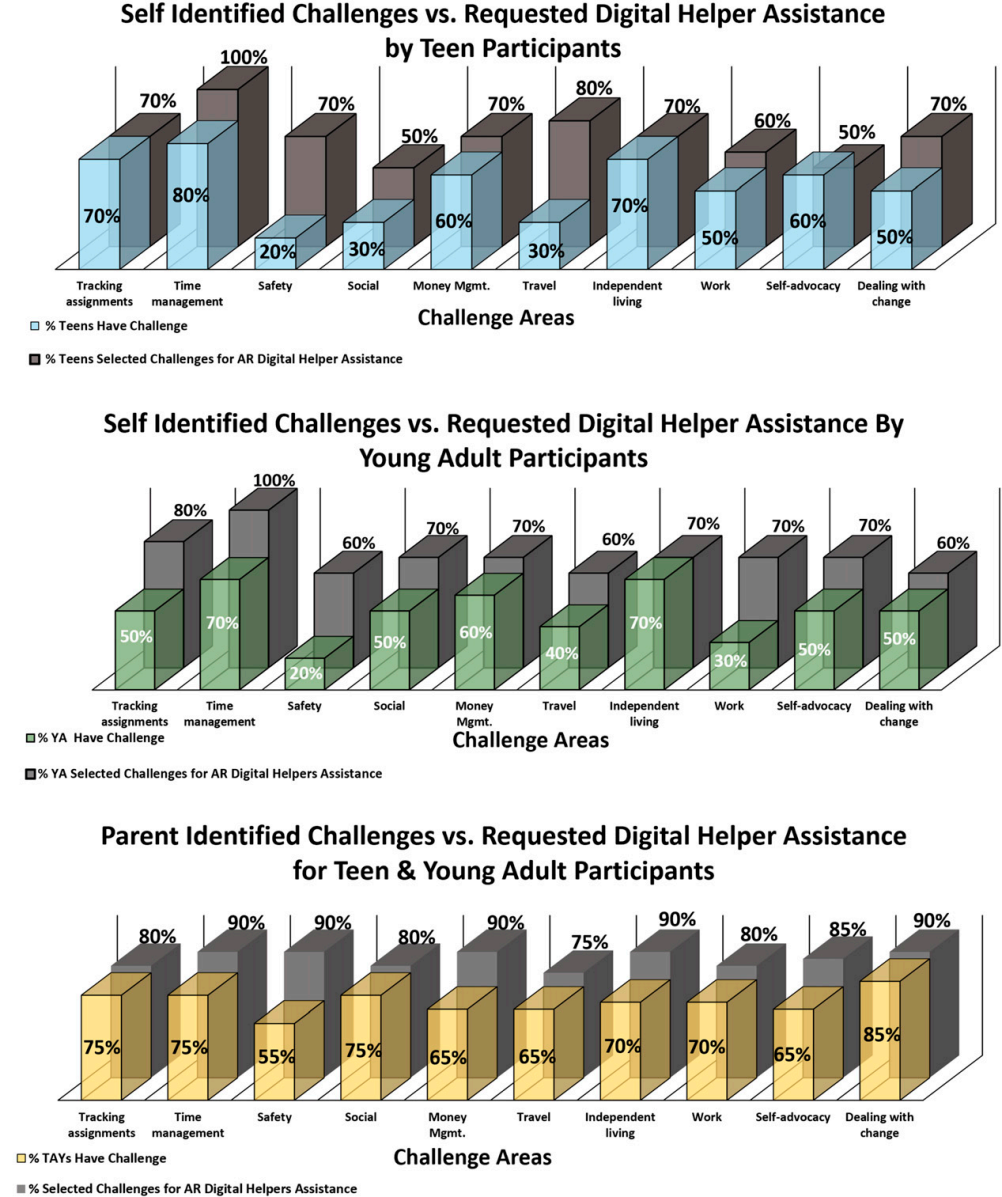
Adolescent, YA, and parent comparisons: challenges vs. areas with which AR digital helpers could assist.

In the area of life challenges,^1,3,27,28^ the study compared what TAY participants and their parents identified as challenges, based on demographic data collected at the first study visit, with the areas where they felt an AR digital helper should offer support. The latter data were collected after viewing the helper in action. Figure 5 shows this comparison. Notably, participants consistently expressed a greater desire for assistance than the percentage who reported having the challenge.

**FIG. 6.**
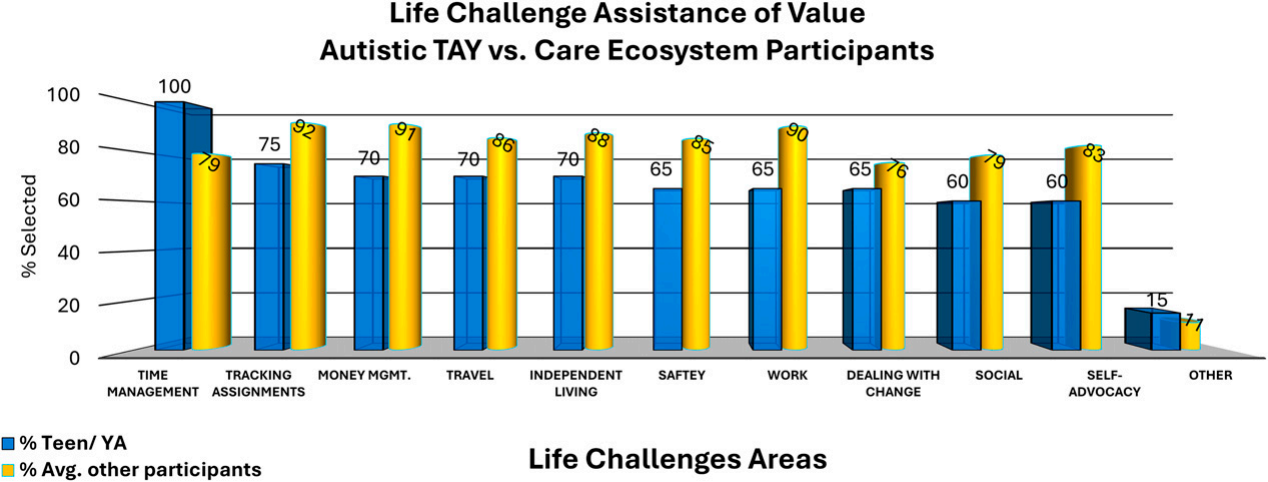
Care ecosystem selects most at higher rate than TAYs digital helper assistance.

These data also reflected that a higher percentage of parents consistently suggested that digital helpers could help their TAYs than the TAYs themselves. This was true for all categories other than time management, which received the only selection by all TAY. Placing the parents in the care ecosystems with the Inv-CP and the Ind-CP and averaging these responses, the trend is identical; all but time management received higher selections as areas of selection for assistance. The data also reflected that parents were more likely than TAYs to suggest that digital helpers could provide support. This trend held across all categories except time management, which was the only area selected by all TAY participants. When responses from parents, Inv-CPs, and Ind-CPs were averaged, the same pattern emerged: all categories except time management received higher ratings for desired assistance. Figure 6 presents this summary.

**FIG. 7.**
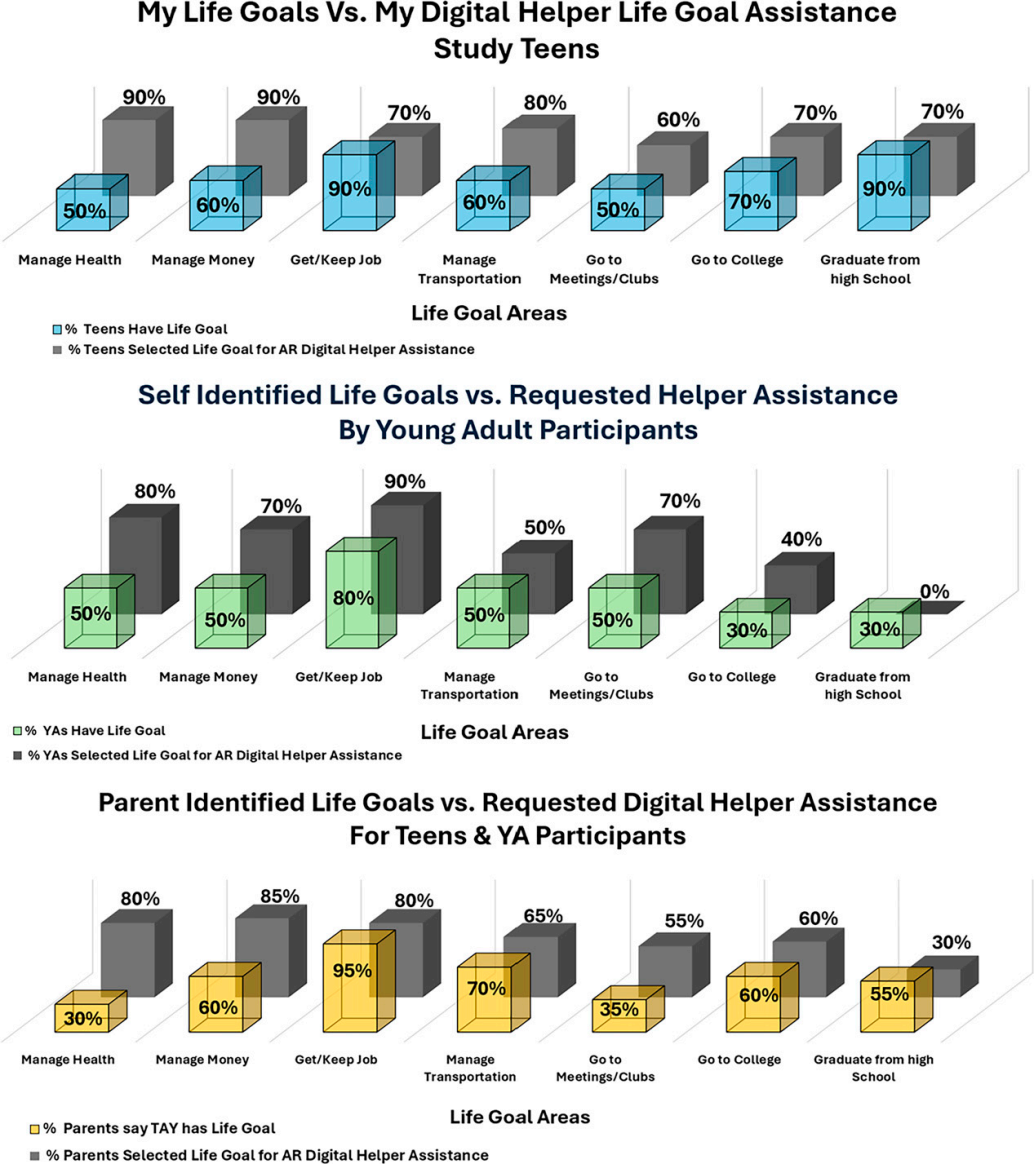
Adolescent, YA, and parent comparisons: life goals vs. areas with which AR digital helpers could assist.

In the area of digital helpers offering support for life goal challenges,^1,3,27,28^ the collected data reflected a similar pattern as Life Challenges support, as is reflected in Figure 7. In the area of life goal challenges, the data reflected a similar pattern, as shown in Figure 7. Most support areas were selected more frequently than the corresponding reported challenges. Exceptions included getting and keeping a job for adolescents and finishing high school for young adults. These results are understandable, given that jobs are less relevant for adolescents aged 14–17 and some young adults may have already completed high school. Still, these areas remain promising for deeper research and understanding.

**FIG. 8.**
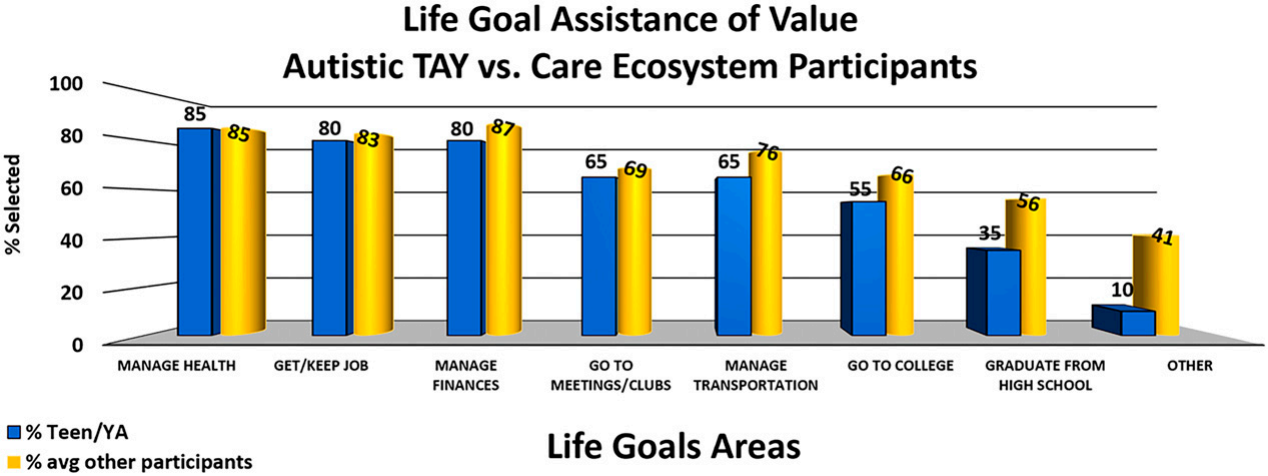
Care ecosystem selects most at higher rate than TAYs for digital helper assistance.

The care ecosystem participants rated the potential of AR digital helpers slightly higher than TAYs in most categories. This is reflected in Figure 8.

Across both life challenges and life goals, all participant groups consistently gave high ratings to the potential utility of AR digital helpers as designed in this study.

### Results: Subquestions

The study also explored three subquestions. First, participants were asked whether giving autistic TAY a codesign role was important. This tested the study’s foundation in inclusive design and participatory research. Only one autistic TAY said they did not care and just wanted assistance. Overall, 96% of participants said codesign was either “very important” or “somewhat important.” Qualitative feedback, especially from Ind-CPs, emphasized codesign’s value for self-esteem. TAY participants said it would be a source of pride.

The second question asked whether AR digital helpers could help autistic TAY reduce life challenges or better achieve life goals. Responses were nearly unanimously “yes,” with only one adolescent selecting unsure, and one selecting “no.”

The third question asked whether AR digital helpers would be good or healthy for autistic TAY to use. All participants responded positively. This section also gathered feedback on concerns about AR digital helpers. Parents expressed the most concern, with 60% citing safety risks related to focus and potential overdependence. The top concern from autistic TAYs was their device running out of battery while using the digital helper.

## Discussion

Using AR as assistive technology for autistic individuals continues to be novel and understudied. This study’s early dataset offers knowledge and indicators of future critical research. Prior to the completion of this study, Lorenzo et al., in 2022, found fewer than 90 ASD research articles that identified use of AR.^13^ These studies focused mainly on using AR markers within articles, books, or game boards for skill practice in areas of safety and social behavior practice and on learning to identify facial gestures.^29–31^ During these years, Sahin et al. established early research using AR glasses with the ASD population to practice identifying facial gesture meanings.^32^ These research efforts were focused on practice and observation rather than assistive support to an autistic individual in their daily lives. No study had focused on this type of assistance using AR until this study.

Since this study, the research on the use of AR for autistic individuals has seen a modest expansion. El Shemy et al. reviewed 44 studies and found that AR-enhanced interventions improved attention, emotional engagement, and pragmatic language use in autistic children.^33^ The use of AR to support the recognition of facial expressions and social cues has continued as a focus in the research.^34^ The Singh and Singh research^35^ tested a deep learning-based AR system used to recognize repetitive gestures and facial expressions as a rare study that has tied AR and AI together in ASD research.

Every participant in this study had lived experience with autism, worked with autistic individuals, or was an autism researcher. The findings support the value of inclusive codesign and show that AR digital helpers can provide effective JIT support across multiple life domains, especially for autistic TAY with IQs above 70.

Prior research using XR mainly used VR and 3D gaming and focused on teaching, practice, or using technology to distract, soothe, or study autistic individuals. This study introduced a real-world assistive intervention for everyday life challenges. It shared the design of the helper with autistic TAY who was the target end user of the helper.

The study established 21 working AR digital helper application prototypes, one for each TAY and one more for all other participants to view in action. These ran on consumer-grade Android devices, demonstrating the feasibility of this type of assistive technology with current consumer devices.

The study’s DCPs aimed to identify the most and least selected characteristics and activities, both before and after participants viewed an AR digital helper in action. This boundary object^24–26^ enabled comparisons across participant types and age groups, including adolescents vs. young adults and TAY vs. their parents and care providers.

Due to the wide spectrum of autism and the study’s inclusive design goals, standard deviation was not used in the analysis. Instead, the study focused on frequency-based comparisons.

The study collected a large volume of data, detailed in the full write-up.^15^ One key finding was that all TAY participants preferred a visible character over a voice-only helper like Alexa© after seeing their codesigned helper. Although the study emphasized that helpers could take any form—or none—85% chose humanoid characters. Of those, 70% reflected specific people such as actors, teachers, or game characters. These were requested twice as often as look-alikes of the TAY themselves.

TAY participants also wanted more complex personalities than the study offered. Many asked for a “happy and…” personality type. Eye contact control and invisibility—features that would allow users to hide their use of a helper—were rated lowest in importance. In contrast, 90% of TAY wanted their helper integrated with their electronic calendar, suggesting a desire to embed the tool into daily life.

There were age-based differences in what participants valued. Young adults prioritized how the helper offered assistance. Adolescents focused more on the helper’s appearance, voice, and personality. While activity preferences varied slightly by age, all TAY participants selected time management as a key area for support. Most also selected tracking assignments, managing health, and gaining or keeping employment.

A notable finding was that participants often selected support areas even when they did not report having that challenge. This suggests strong perceived value in AR digital helpers. Qualitative feedback from all groups highlighted the potential for emotional and motivational support. New use cases from TAY participants included that they could use a helper to manage anger, offer someone to talk to, or provide voice volume cues for social situations.

All three of the study’s three subquestions (value of codesign, utility, and whether helpers would be healthy for autistic TAY) received overwhelmingly positive responses. Concerns were also important to capture. Parents and care providers raised issues around distraction and overdependence. They recommended features like visibility toggles or contextual deactivation to reduce risks. These insights underscore the need for flexible, customizable tools that adapt to individual needs and levels of independence.

The study had a number of limitations. While the participant count was larger than typical XR studies in the autism to date, a significantly larger follow-up study is needed. The research focused on a specific subset of autistic TAY and high IQs, while the researchers believe such a tool might have a greater impact for individuals with lower IQs. To address COVID-19 restrictions, each codesigned helper was filmed in first person to simulate real use. These videos served as boundary objects^24–26^ to help participants understand the helpers. The prototype supported only three assistive activities. To reduce this limitation, the selected tasks, travel independence, executive functioning, and wayfinding, were chosen to reflect broad potential. All prototypes, data collection, and analysis were conducted by a single researcher as part of doctoral research. Although the study was reviewed and approved by IRBs at two universities and supported by a full advisory committee, further research by additional teams is essential to expand our knowledge. This study also concluded just before the rise of research-ready Generative AI (GenAI) tools. Adding GenAI holds particular promise for advancing this line of research and accelerating innovation across assistive technologies. GenAI’s strength in supporting adaptive, context-aware interactions that are inclusively curated with less predetermined dialog, due to its natural language processing, promises research that able to support more personalized and empowering digital experiences.

## Conclusions

This study establishes a foundation for applied AR JIT digital helpers for autistic individuals, demonstrating inclusive codesign feasibility using commercial components. Response, especially from autistic TAY and their parent participants, was overwhelmingly positive, with some concerns noted around safety and sensory factors. The technology’s near-term viability and strong participant interest underscore its promise. Future research should expand to other population segments and integrate external systems to enhance safety, personalization, and cognitive support. The primary researcher is now advancing GenAI-enhanced prototypes of avatar-based digital helpers with clinician input to further personalize and scale these assistive tools.

## Abbreviations Used

AI: Artificial Intelligence
AR: Augmented Reality
ASD: Autism Spectrum Disorder
CDC: Center for Disease Control and Prevention
COVID-19: Coronavirus Disease 2019
DCP: Data Collection Protocol
DMS-5: Diagnostic and Statistical Manual of Mental Disorders, Fifth Edition
GenAI: Generative Artificial Intelligence
IDT: Inclusive Design Theory
Ind-CP: Independent Care Provider
Inv-CP: Involved Care Provider
IQ: Intelligence Quotient
IRB: Internal Review Board
JIT: Just-In-Time
TAY: Transition Age Youth
VR: Virtual Reality
XR: Extended Reality
YA: Young Adult
